# Double-counting of populations in evidence synthesis in public health: a call for awareness and future methodological development

**DOI:** 10.1186/s12889-022-14213-6

**Published:** 2022-09-27

**Authors:** Humaira Hussein, Clareece R. Nevill, Anna Meffen, Keith R. Abrams, Sylwia Bujkiewicz, Alex J. Sutton, Laura J. Gray

**Affiliations:** 1 Department of Health Sciences, University of Leicester, University Road, Leicester, LE1 7RH UK; 2 Department of Statistics, University of Warwick, Coventry, CV4 7AL UK

**Keywords:** Evidence synthesis, meta-analysis, Network meta-analysis, Double-counting, Real-world data

## Abstract

**Background:**

There is a growing interest in the inclusion of real-world and observational studies in evidence synthesis such as meta-analysis and network meta-analysis in public health. While this approach offers great epidemiological opportunities, use of such studies often introduce a significant issue of double-counting of participants and databases in a single analysis. Therefore, this study aims to introduce and illustrate the nuances of double-counting of individuals in evidence synthesis including real-world and observational data with a focus on public health.

**Methods:**

The issues associated with double-counting of individuals in evidence synthesis are highlighted with a number of case studies. Further, double-counting of information in varying scenarios is discussed with potential solutions highlighted.

**Results:**

Use of studies of real-world data and/or established cohort studies, for example studies evaluating the effectiveness of therapies using health record data, often introduce a significant issue of double-counting of individuals and databases. This refers to the inclusion of the same individuals multiple times in a single analysis. Double-counting can occur in a number of manners, such as, when multiple studies utilise the same database, when there is overlapping timeframes of analysis or common treatment arms across studies. Some common practices to address this include synthesis of data only from peer-reviewed studies, utilising the study that provides the greatest information (e.g. largest, newest, greater outcomes reported) or analysing outcomes at different time points.

**Conclusions:**

While common practices currently used can mitigate some of the impact of double-counting of participants in evidence synthesis including real-world and observational studies, there is a clear need for methodological and guideline development to address this increasingly significant issue.

## Introduction

Both in the evaluation of health technologies and epidemiological research, systematic reviews and meta-analysis are regarded as providing high quality evidence [1, 2]. With the heightening interest in studies reporting the use of real-world data in health research literature, which include observational studies using registry or electronic health record data collected routinely in clinical practice, the incorporation of these studies in evidence synthesis is becoming increasingly common [3–6]. Utilising data from all available sources, including observational studies, can provide many benefits in epidemiology, such as increased power and more generalizable results. However, this can often introduce a number of analytical problems such as confounding, significant heterogeneity and misclassification bias within the non-randomised evidence.

While methods such as meta-regression have been considered to address these issues [7, 8], a significant problem that has received little attention within public health research is the double-counting, also referred to as sample overlap, of individuals and databases when including such studies in evidence syntheses. With the increased use of cohort and real-world data in evidence synthesis, double-counting has the potential to become a significant issue. Some aspects of double-counting have been discussed by Senn (2009) and Lunny et al.,(2021), specifically in the context of whole studies or study arms which were being included multiple times in the meta-analysis [9, 10]. More attention has been given to this issue in the fields of social science, education, economics and finance, where analytical approaches to dealing with such issues have been suggested [11, 12]. However, currently there is no published guidance available on how to address this.

Sample overlap between studies will lead to spuriously high precision in meta-analysis and is also potentially a source of bias. Due to this, many reviewers choose to exclude or adjust for studies where there is an overlap of participants. It may not be obvious if studies contain overlapping patients and so double-counting of individuals in a synthesis may exist without the reviewer’s knowledge. Whilst guidance documents for conducting systematic reviews and meta-analysis of intervention and prevalence/incidence studies exist, none of these consider the effect of the large magnitude of sample overlap expected in whole population studies on meta-analysis results [13, 14]. Therefore, this paper aims to highlight and illustrate some of the specific methodological and practical aspects of double-counting of individuals and datasets that should be considered in evidence syntheses that include real-world and observational data using a number of public health case studies.

## Methods

Various aspects of double-counting of populations in included observational and real-world evidence studies in meta-analysis, henceforth referred to as the overlapping populations problem for brevity, will be discussed with case-studies used to highlight particular issues. The case studies used to illustrate the common issues with overlapping populations have been identified through involvement of authors of this manuscript with multiple applied synthesis projects [15–18]. Each case study has utilised various approaches of addressing the problem; in each scenario the method used to address this issue by authors was discussed, which considered the most appropriate approach to answer the research question and maximise the data available. Further aspects of overlapping populations in specialised areas of health research, that have the potential to become important issues, are then considered. The implications for research will be discussed and potential methods to address these issues reviewed.

## Results

### Case study 1 – COVID-19 outcomes by ethnicity

This case study considers a systematic review and meta-analysis which aimed to assess the effect of ethnicity on a range of COVID-19 outcomes and faced multiple issues regarding double-counting [18]. Three examples of double-counting of individuals/populations that were highlighted by the authors in this study are summarised below (details obtained from contact with the author group):

1. Multiple studies using same research database:

Four studies [19–22] were identified which had used data from UK Biobank, a consented cohort study, and presented data on ethnicity differences and COVID-19 infectivity. To address the issue of overlapping populations the authors included only one of the four studies, favouring peer reviewed papers over pre-prints and then choosing the largest study:

- 16th March–14th April 2020, 669 COVID-19 positive cases from entire UK Biobank sample (502,536 participants) [Pre-print] [19]
- 16th March–3rd May 2020, 948 COVID-19 positive cases from reduced UK Biobank sample (392,116 participants) [Peer-reviewed] [20]
- 16th March–14th April 2020, 651 COVID-19 positive cases from reduced UK Biobank sample (415,582 participants) [Pre-print] [21]
- 16th March–14th April 2020, 669 COVID-19 positive cases from entire UK Biobank sample (502,536 participants) [Pre-print] [22]

**Fig. 1:**
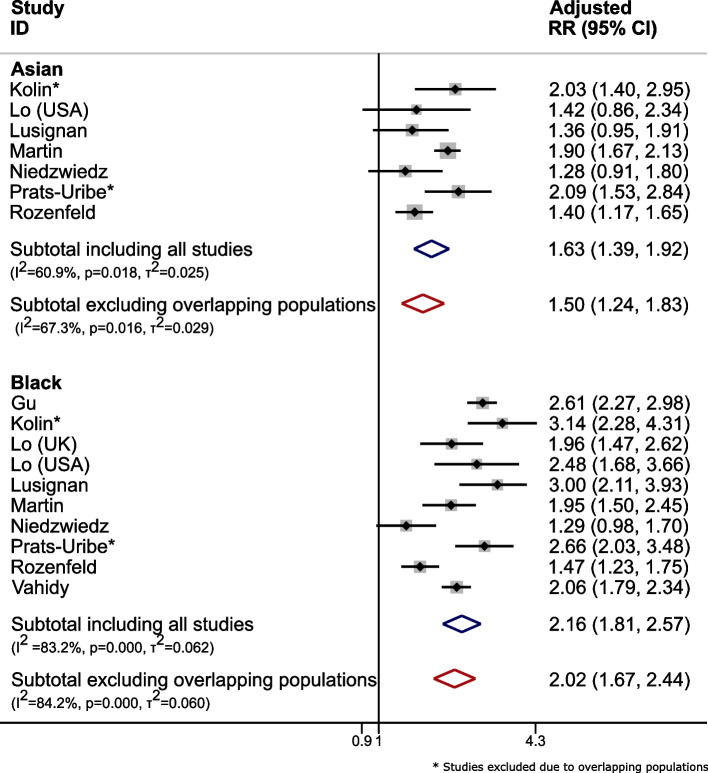
Forest plots for meta-analysis of adjusted RR for infectivity of COVID-19 in people with Asian or Black ethnicity versus those of White ethnicity (Blue: including all eligible studies; Red: after removal of studies with overlapping populations) in case study 1. Note: Z Raisi-Estabragh et al., [20] is not included in the figure as they did not report adjusted RR

Hence, only Niedzwiedz et al.,(2020) [20] was included. Figure 1 reports the adjusted risk ratios before and after excluding overlapping populations. There were reductions in overall relative effect estimates, with increased heterogeneity estimates after excluding overlapping population papers. For example in the Asian population, risk ratio (RR) of infectivity of COVID-19 reduced from 1.63(1.39,1.92) to 1.50(1.24,1.83) with increased uncertainty.

(2) Multiple studies using the same hospital database
  Five eligible studies [23–27] were identified which had used data from the Mount Sinai Health System, New York. Two of these assessed specific subgroups; (i) patients with HIV [24] and (ii) patients with myeloma [26], which were excluded as these patient subgroups would be included in the other three papers which assessed all those with COVID-19 in the database. The remaining three papers each included slightly different subsets of the same hospital cohort:
  27th Feb–2nd April 2020, *n* = 2199 hospitalised patients with COVID-19 [23]
  24th Feb-15th April 2020, *n* = 3273 hospitalised patients with COVID-19 [27]
  Up to 15th April 2020, *n* = 7592 confirmed COVID-19 patients (inpatient, outpatient and emergency) [25]

As the dates for all three studies overlapped significantly and all included hospital inpatients, the largest study was included [25], which also gave the most complete data.

**Fig. 2:**
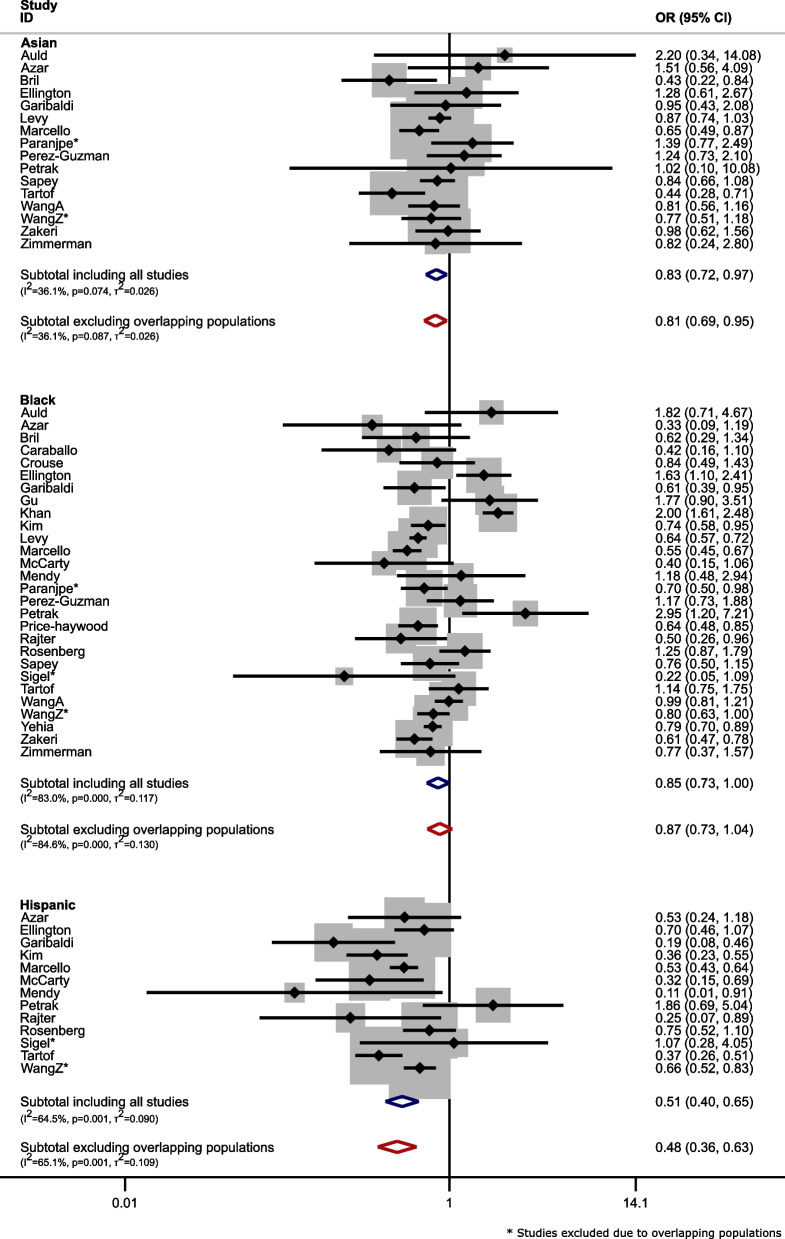
Forest plots for meta-analysis of odds ratios (OR) for mortality after contraction of COVID-19 of people with Asian, Black, or Hispanic ethnicity versus those of White ethnicity (Blue: including all eligible studies; Red: after removal of studies with overlapping populations) in case study 1. NOTE: B. Wang et al., [24] is not included in figure as they did not report the necessary data to calculate the specified unadjusted OR

Figure 2 shows the odds ratios for mortality after contraction of COVID-19, before and after removing overlapping population studies. There were little changes in odds ratios of mortality after contracting COVID-19 before and after removing overlapping population studies; although no difference was seen in this example, there might be cases of larger overlap where this may not have been the case.

(3) Multiple studies from the same country:

The majority of papers included in the review analysed data from the USA (84%). Some studies were nationwide, for example, the Centers for Disease Control and Prevention published data on all women of reproductive age across the USA [28]. Other studies were conducted in the same states but often with no specific information on which hospitals were used. Where overlap, such that a study (ies) population was fully encompassed by another, could not be established, all studies in question were included. In the UK, two studies included UK wide data [29, 30]; only Williamson et al. (2020) [30] was included in the review as it had the larger sample size. Examples [1] and [2] as described above may appear to be more conservative (i.e. exclude all but one study) than the approach described in this section. However, this is a reflection on the general rule-of-thumb that was conducted throughout the meta-analysis; only exclude studies when the overlap was deemed to be ‘certain’, optimising data available.

### Case study 2 – incidence of major limb amputation in the UK

**Fig. 3:**
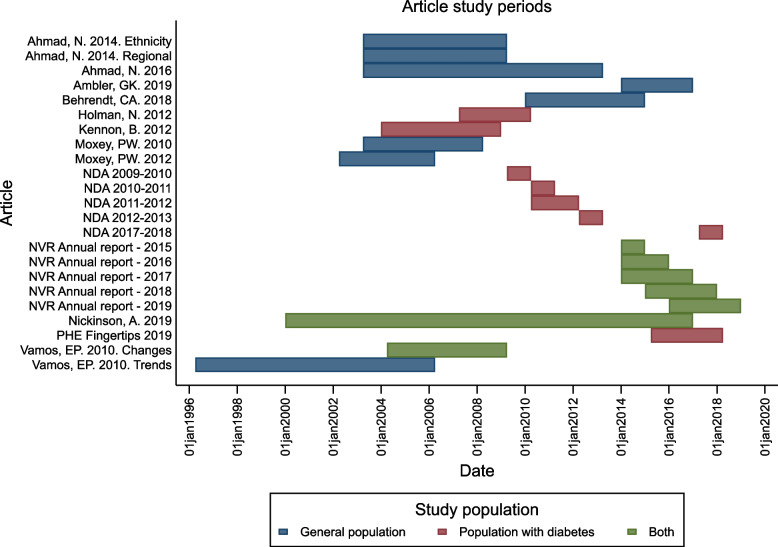
Included article study periods by population type (general population, population with diabetes, reported for both the general population and population with diabetes) in case study 2

When conducting a systematic review on the reasons for variation in reported incidence of major lower limb amputation [16, 17], a major issue was the overlap of study period. Data syntheses including whole country population studies may mean combining studies with a major participant overlap. In addition to the problem of patient overlap due to using the same national health record database, in this review, suspected patient overlap was also observed between studies using the same or similar time periods. Figure 3 illustrates the overlapping of study populations in the included review articles by plotting the study periods for each article. As nearly all articles experienced an overlap in study period and no articles provided the information necessary to gauge the magnitude of the participant overlap, articles were not excluded from the review. Given this overlap (and the heterogeneous methods of the included articles) statistically combining data would not have provided a meaningful outcome; only a narrative summary could be provided.

### Case study 3 – use of SGLT-2is and GLP-1RAs in type 2 diabetes

The previous two case studies have focused solely on synthesis of observational studies. Overlapping of data is also an issue when incorporating real-world evidence into synthesis of Randomised Controlled Trials (RCTs). This case study discusses an extension to include real-world evidence in a previously published systematic review and Network Meta Analysis (NMA) of RCTs of two classes of glucose-lowering medications in type 2 diabetes [15]. While including real-world evidence, a number of issues related to double-counting were highlighted:

1. Multiple studies sharing common treatment arms:

In this NMA, assessing the effect of the treatments on the change from baseline in HbA1c(%) measuring blood-glucose levels, multiple observational studies utilising the same database were identified. Reporting results after 24 weeks of intervention, two US studies had used the Quintiles Electronic Medical Database to evaluate treatment effects [31, 32]. Saunders et al.,(2016) [31] extracted data to compare participants given liraglutide to those given exenatide once weekly (QW) between 1st Feb 2012-31st May 2013. Unni et al.,(2017) [32] compared participants prescribed albiglutide, dulaglutide or exenatide QW between the 1st Feb 2012-31st March 2015. The overlapping treatment arm of exenatide QW over a common timeframe would likely result in some overlapping participants in these two studies. Therefore, sensitivity analysis was conducted by excluding the exenatide QW arm from the study conducted by Unni et al.,(2017) [32] and including all other arms in that study.

(2) Overlapping studies at multiple time points:

In addition to the two studies described above, McAdam-Marx et al.,(2016) [33] conducted a study comparing standard care to liraglutide and exenatide QW users using the Quintiles Electronic Medical Database. However, this study analysed the change in HbA1c(%) after 52 weeks of treatment. It is often desirable to synthesise all time points in the same statistical model because of benefits in efficiency and coherence. While this is not an issue in this particular case study, as multiple time points have been analysed separately, if all data were synthesised in a single analysis, the issue of data overlap would be present. In that case, the study with the longest follow-up would be included in the synthesis.

(3) Overlapping of participants in studies using the same database even when the treatments being investigated are not common:

While there is obvious overlap in participants in studies utilising the same database with a common treatment arm, a more difficult to identify issue of overlapping participants may occur if the treatment arms used are not common between the studies. In this case study, multiple studies utilised the Optum Research database [34, 35]. One study compared the use of albiglutide to liraglutide between July 2014–December 2015 [35] while another compared canagliflozin to dapagliflozin users between January 2014–September 2016 [34]. In both studies, participants could be on background therapies in addition to the study treatment. Therefore, there is a possibility that participants on combination therapy could be included multiple times in the NMA (e.g., participants given liraglutide also given canagliflozin). In this instance, it is difficult to extract the overlapping participants, and therefore all studies were included in the NMA.

### Other potential issues

#### Overlap of real-world evidence and randomised controlled trial data

There are many methods for participant recruitment into a trial; some methods include referrals from clinicians and/or physicians, electronic health records and from registries [36, 37]. For example, in the UK Collaborative Trial of Ovarian Cancer Screening (UKCTOCS) trial, more than 200,000 participants were recruited from 13 UK registries to be randomised to various screening methods [38, 39]. However, recruiting patients from these settings will mean there is a possibility of duplicated participants from the observational and RCT data in the evidence synthesis. Using the example from the UKSTOCS trial, if this trial is included in the evidence synthesis, along with an observational study using at least one of the recruitment registries for UKCTOCS, there is a high likelihood of participants overlapping in the RCT and observational study.

This issue of duplicated participants in RCT and observational data is particularly apparent in the Systematic Anti-Cancer Therapy (SACT) database [40]. As SACT include all patients in the UK to be given anti-cancer therapy, those in RCTs receiving particular therapies would also be included in the SACT data, resulting in participants being duplicated if combined in a meta-analysis.

#### Single arm studies and studies with historical controls

There are increasing numbers of single arm trials conducted for regulatory purposes to assess effectiveness of interventions. These are often designed to compare treatment effectiveness in participants before and after interventions [41]. Alternatively, single-arm data are sometimes compared to historical controls (control arm data from a past study) [41]. To get reliable historical control arms, groups are collating control arms from certain past studies to generate databases for specific areas of disease research [42]. Therefore, single-arm studies that access the same control databases may use the same set of participants for recruitment of historical control data. Including multiple single-arm studies with historical controls in an evidence synthesis analysis could result in the duplication of participants in the control arms.

#### Multicentre studies

Duplicate/multiple publication has been defined as the “publication of a paper that overlaps substantially with one already published”–so called ‘salami slicing’ [43]. This needs to be taken into account in both RCTs and observational studies. Sometimes individual sites from multicentre RCTs publish results separately without cross-referencing the main analysis [44]. Involving all publications in evidence synthesis could result in overlapping participants, with poor reporting leading to confusion on whether participants have been recruited for the larger RCT or a specific centre [44]. Further, multiple publication could also be an issue in observational studies [45]. In England, provided researchers obtain consent and ethical approval, individual hospitals and regions can publish cohort studies. However, data could also be collected for larger health care databases such as NHS Hospital Episode Statistics Admitted Patient Care data, which collects information from 451 NHS trust hospitals in England [46]. When collecting the totality of evidence for a systematic review, if the link between publications is not made clear by the authors of the primary studies, individuals could be duplicated when conducting the evidence synthesis.

## Possible solutions

It is important to extract the pertinent information for studies included in order to make an informed decision on which studies/treatment arms to include in the evidence synthesis when including observational studies (Box 1). An essential step would be to determine what information can be clarified by contacting authors of the primary studies, such as database used or timeframe of analysis if unclear. Alternatively, obtaining anonymised patient IDs and/or Individual Patient Data (IPD) of those analysed could be used to determine the percentage overlap across studies utilising and excluding duplicate individuals from the same real-world data. However, IPD can often be difficult or expensive to obtain and may not always be feasible in a meta-analysis study.

Box 1 Suggested approaches to include real-world data in evidence synthesis Identify potential overlapping populations by extracting data on:

- Where the data is from:
  - ○ Database or registry used
  - ○ Hospital (and if possible specific department(s) data is from)
  - ○ Geographical area(s)
- Time period of study
- Population characteristics (e.g., age range, background interventions or particular subgroup considered).

Options to minimise impact of double-counting of individuals/populations:

- Consider using a method of analysis which accounts for double-counting
- Contact authors to clarify aspects of the studies that are unclear
- Include all studies if double-counting cannot be fully determined
- Analyse studies at different time-points
- Preference of peer-reviewed studies
- Retain only one of any identified set of studies in which overlap is suspect by some rational criteria. For example, retain the:
  - ○ Largest study (i.e., study with the most participants)
  - ○ Most recent study
  - ○ Most complete data
  Authors could utilise an alternative study if the selected study does not have data for a particular outcome being analysed
- Obtain individual patient data
- Always conduct sensitivity analysis to assess robustness of results.

NOTE: The authors are not recommending these approaches rather highlighting possible options; further work is required to understand the implications of these methods.

Reporting on approaches taken:

- Provide rationale for studies included in the evidence synthesis
- Discuss potential double-counting of data between studies
- Implications of double-counting and method used to account for it regarding interpretation of results.

In the case of observational studies carried out using different subsets of participants from the same database, all studies could be included provided there is no overlap of intervention arms, background therapies and/or timeframe of analysis across the included studies. Alternative solutions that have been utilised in the case studies described above included preference of peer-reviewed studies (vs pre-print) and larger studies to be included in the evidence synthesis. Alternatively, the most recent or comprehensive study of those that used the same database could be considered. If multiple studies in the evidence synthesis are conducted at different time-points (i.e. outcome follow-up time differ), as in case study 3, the analysis could be conducted at the separate time-points.

Another solution, which was not considered by any of the included case studies, is to include all data regardless of overlap within the synthesis, but use a statistical method which accounts for these dependences. One such method is that proposed by Bom and Rachinger (2020) which suggests a generalized- weights meta estimator which deals with this issue by modeling the variance-covariance matrix that describes the structure of dependence among estimates [11]. Although the authors state that this method is ‘fairly straightforward to implement’ it does require data on the number of overlapping observations – which may not be possible to estimate in many scenarios, such as the case studies used here [11]. Methods which have been proposed for dealing with clustering of effect estimates in meta-analyses may also be useful here, but are yet to be evaluated in this setting [12].

## Discussion

The use of real-world data in evidence synthesis within public health is becoming increasingly common, and provides a number of benefits from an epidemiological standpoint, such as increased power and greater generalisability. However, double-counting of individuals across studies is becoming a significant problem, particularly in meta-analyses; currently, there is no guidance to account for this issue in meta-analysis. In this paper, the issue of double-counting of overlapping populations was discussed in reference to a number of case studies. However, it should be noted that while case studies have been used to illustrate the issues with double-counting of individuals/populations, this is not to be overly critical of the work reported in these papers and may not reflect all potential issues.

In the case studies, while double-counting of populations/individuals is considered an important issue, it has not been adjusted for in a meta-analysis in any detailed manner apart from using standard approaches [18]. Inclusion of larger studies or those that provide a greater level of relevant information (e.g. more outcome data or more complete population) is a possible solution in order to minimise the impact of overlapping populations. If data are unavailable in the most comprehensive study for an outcome of interest, an alternative study could be utilised [18]. However, excluding studies could also result in non-duplicated participants being excluded, leading to a potential loss of power in the analysis. It may be possible to use IPD to address some of the issues, which may also mitigate the issue of power loss; however, in many cases, for example UK Biobank data and the Clinical Practice Research Datalink (CPRD), data must be destroyed after a certain period of time and cannot be shared. Further, as many databases contain anonymised data, it would be difficult to identify participants duplicated across multiple datasets. Proposed analytical methods for dealing with study overlap were not considered [11], although the requirement for data on the extent of the overlap would have been challenging to estimate. All potential solutions considered have benefits and limitations. For example, given known issues with publication bias prioritising peer reviewed articles above pre-prints may not always be sensible. Without further methodological research comparing methods under different scenarios (e.g. size of overlap, number of studies with overlap) it is impossible to give strong recommendations. Therefore, it is vital to carry out sensitivity analyses, excluding studies in which duplicate participants may be a concern to assess if results are robust. As seen in the case studies, evidence syntheses utilise different approaches to address overlapping populations; therefore, recommendations based on robust evaluations of available methods are needed for a standardised method of addressing this issue.

In order to assess the potential for double-counting when performing evidence synthesis, researchers rely on the reporting quality of the original studies. Often it is not possible to determine if overlap is an issue or the extent of it. The REporting of studies Conducted using Observational Routinely-collected Data (RECORD) collaborative have developed reporting guidelines for studies using real-world data [47]. This is an extension to the STrengthening the Reporting of OBservational studies in Epidemiology (STROBE) reporting guidelines which cover all observational studies [48], including additional recommendations specific to the use of routinely collected data. It is hoped that such guidelines will improve the reporting quality of studies using real-world data so that the extent of overlap can be considered. Even with transparent reporting of data provenance, the risk of double-counting can never be completely removed, but it can be minimised.

There are a number of serious consequences when double-counting of information is not taken into account in evidence synthesis. Including overlapping populations can artificially inflate the precision of effect estimates, potentially leading to inappropriate conclusions drawn from the analysis and consequently inappropriate decisions made based on such data [11]. Sensitivity analysis conducted in case study 1 showed less certainty around relative effect estimates after excluding overlapping population studies with increased heterogeneity. While some changes to effect estimates were minimal, this issue could prove to be much larger in other areas and have an impact in evidence-based decision-making when, for example, such estimates are used as inputs into a cost-effectiveness analysis. Additional issues as a consequence of overlapping populations include duplicate publication bias [14]. This refers to the bias introduced when individual centres from a multicentre study publish results but do not specify that it is from a multiple centre study. The same bias can be extended to real-world studies where hospital data are utilised in a region, but the specific hospital from which the data is derived from is not mentioned; this makes it difficult to distinguish the amount of overlap across the various studies in the evidence synthesis model and so increasing the possibility of bias. Further methodological research needs to be undertaken to assess the full impact of this issue as well as development of appropriate methodologies to address it.

## Conclusions

This manuscript has highlighted a number of issues and challenges associated with double-counting of individuals and databases when including real-world data in meta-analysis within the field of public health. However, current approaches to address this issue may result in relevant available information not being fully utilised, leading to a potential reduction in analysis power. This manuscript calls attention to the need for clear guidance and methodological development, to ensure appropriate and efficient inclusion of real-world and observational data in evidence synthesis, without increasing bias or spurious precision while making maximal use of the data available.

## Data Availability

No new data were generated or analysed in support of this research. The study includes no data from unpublished restricted [non-publicly available] human clinical databases. The data that support the findings of this study are available from the corresponding author upon reasonable request.

## Abbreviations

RCTs: Randomised Controlled Trials
NMA: Network Meta Analysis
QW: Once weekly
UKCTOCS: UK Collaborative Trial of Ovarian Cancer Screening
SACT: Systematic Anti-Cancer Therapy
IPD: Individual Patient Data
CPRD: Clinical Practice Research Datalink
