# Publisher Correction: Double-counting of populations in evidence synthesis in public health: a call for awareness and future methodological development

**DOI:** 10.1186/s12889-022-14741-1

**Published:** 2022-12-08

**Authors:** Humaira Hussein, Clareece R. Nevill, Anna Mefen, Keith R. Abrams, Sylwia Bujkiewicz, Alex J. Sutton, Laura J. Gray

**Affiliations:** 1 Department of Health Sciences, University of Leicester, University Road, Leicester, LE1 7RH UK; 2 Department of Statistics, University of Warwick, Coventry, CV4 7AL UK

**Publisher Correction: BMC Public Health 22, 1827 (2022)**

**https://doi.org/10.1186/s12889-022-14213-6**

In the original publication of this article [1]: Box 1 was omitted during the publication process. Box 1 has been included in this correction article, the original article has been updated.

Box 1 Suggested approaches to include real-world data in evidence synthesis Identify potential overlapping populations by extracting data on:

- Where the data is from:
  - ○ Database or registry used
  - ○ Hospital (and if possible specific department(s) data is from)
  - ○ Geographical area(s)
- Time period of study
- Population characteristics (e.g., age range, background interventions or particular subgroup considered).

Options to minimise impact of double-counting of individuals/populations:

- Consider using a method of analysis which accounts for double-counting
- Contact authors to clarify aspects of the studies that are unclear
- Include all studies if double-counting cannot be fully determined
- Analyse studies at different time-points
- Preference of peer-reviewed studies
- Retain only one of any identified set of studies in which overlap is suspect by some rational criteria. For example, retain the:
  - ○ Largest study (i.e., study with the most participants)
  - ○ Most recent study
  - ○ Most complete data

Authors could utilise an alternative study if the selected study does not have data for a particular outcome being analysed

- Obtain individual patient data
- Always conduct sensitivity analysis to assess robustness of results.

NOTE: The authors are not recommending these approaches rather highlighting possible options; further work is required to understand the implications of these methods.

Reporting on approaches taken:

- Provide rationale for studies included in the evidence synthesis
- Discuss potential double-counting of data between studies
- Implications of double-counting and method used to account for it regarding interpretation of results.
